# Between nature and culture – Interpreting students’ sexuality in physical education

**DOI:** 10.3389/fsoc.2024.1374488

**Published:** 2024-03-20

**Authors:** Nicola Böhlke, Benjamin Zander, Daniel Rode

**Affiliations:** ^1^Institute of Sport Science and Movement Pedagogic, Technische Universität Braunschweig, Braunschweig, Germany; ^2^Institute of Sport Science, University of Göttingen, Göttingen, Germany; ^3^Department of Sport and Exercise Science, Paris Lodron University Salzburg, Salzburg, Austria

**Keywords:** Sexuality, physical education, online research, discourse analysis, interpretive schemes

## Abstract

**Introduction:**

As sexuality in physical education (PE) is often treated as a taboo subject, social media platforms, online chats, and internet forums are emerging as spaces where it is negotiated more openly and broadly by current and former actors of the field. This paper contributes to a better understanding of the discursive construction of sexuality in PE in such online communication.

**Methods:**

In line with The Sociology of Knowledge Approach to Discourse (SKAD) we investigate basic schemes of interpretation of sexuality with a heterogeneous sample of threads (17 threads from seven different online forums) on different PE situations in Germany. The threads are analyzed using grounded theory coding procedures.

**Results:**

Our discourse analysis reveals that the multifaceted and often controversial online discussions are structured by two dominant schemes of interpreting students’ sexuality in PE, both of which are differentiated in complex ways: The online communication draws on – and by that reproduces – a nature and a culture perspective on constellations of body, sex, gender, and sexuality. We detail how from each perspective, different knowledge about these constellations, different everyday phenomena and problems in PE, and different norms for dealing with these phenomena and problems become important.

**Discussion:**

Discussing these results in the context of previous literature, we argue that it is important to address sexuality in a subject-specific approach and take the discursive knowledge and fundamental schemes of interpretation into account that shape the (im-)possibilities of addressing sexuality in PE.

## 1 Introduction

Physical education (PE) has long been discussed as a school subject that is inseparably linked to issues of the body such as health, performance, gender, or dis−/ability (Kirk et al., 2006). In this context, sexuality is a phenomenon that has often been neglected (Clarke, 2006). Yet, there is a growing body of scholarship that has been establishing sexuality in PE as a field of empirical research and pedagogical consideration (Landi, 2019a; Van der Steeg et al., 2021; Varea and Öhman, 2022), with current studies emphasizing specific focuses, such as queerness or sexuality education, but devoting less consideration to the phenomenon of sexuality in PE in general. The previous scholarship demonstrates that aspects of sexuality, including sexualized boundary violations, physical contact, queerness, desire, and their association with matters of body and gender, have a notable impact on teachers’ and students’ experiences of PE. While this is often treated as a taboo subject, social media platforms, online chats, and internet forums are emerging as spaces where current and former actors of PE articulate and discuss a broad range of aspects and topics related to sexuality in PE more openly (Böhlke and Zander, 2022). Building on these insights, existing scholarship emphasizes the need of critically interrogating PE as a field for either reproducing or surfacing, challenging, and transforming the “truths,” norms, and power relations that are tied to students’ sexuality in this field.

In this paper, we argue that gaining a better understanding of how students’ sexuality is interpreted in PE-related discourses is crucial for this task. Following a discourse analytical perspective, we regard PE as a field that is permeated by various discourses. As practices that systematically produce the objects of which they speak (Foucault, 1983), these discourses provide specific knowledge and “truths” about students’ sexuality that help shape realities and experiences of PE. Students and teachers are, however, not passively at the mercy of such discourses. Rather, they (re-)produce, contribute to, and negotiate them actively – for instance in emerging internet spaces. According to a sociology of knowledge approach to discourse, these negotiations are structured by fundamental schemes of interpretation (Keller, 2013, 2018). This means that the diverse discussions in internet forums draw on underlying patterns of how the sexuality of students can be contextualized, referred to, valued, and interpreted so that students, their bodies, and their behavior can become intelligible within PE.

Our paper draws on a qualitative discourse analysis of 17 threads from 7 different internet forums in which the sexuality of students in PE is discussed by users that, in their communication, identify as actors related to PE. Our purpose is to answer the following research question: Which schemes of interpretation structure the discussions of students’ sexuality in PE in these internet threads? Answering this question, our study provides a better understanding of the discursive production of the norms, subjectivities, and power relations that are tied to the phenomenon of students’ sexuality in PE.

## 2 Materials and methods

### 2.1 State of research: sexuality in PE

In PE research, sexuality presents a marginal research topic that has mostly been investigated in the context of research on bodies, gender, and heteronormativity in PE, and specifically in LGBTIQ research. In these lines of research, gender is emphasized as a relevant category of difference in PE (e.g., Penney, 2002) with studies showing that the actualization of gender takes place in the context of peer affiliations, body-related practices or physical staging practices (e.g., Gorely et al., 2003). Sexuality appears to be closely interwoven with gender constructions (e.g., Clarke, 2006) and can function as a means of exercising violence or as a means of power (Fitzpatrick and Enright, 2016), e.g., in the context of the teacher-student relationship (e.g., Böhlke and Zander, 2022). Existing research on sexuality focusses on specific aspects of sexuality in PE, providing important insights into these aspects while leaving much room for further exploration. Previous work deals with sexualized boundary violations (Gråstén and Kokkonen, 2022; Wagner and Knoke, 2022), interpersonal body contact (Varea and Öhman, 2022) and ethical concepts of positive sexual integrity (van der Steeg et al., 2021). LGBTIQ research explores the experiences of LGBTIQ individuals (e.g., Landi, 2019b; Müller and Böhlke, 2021) or focuses on pedagogical practices and structures of PE (Sykes, 2011; Landi, 2019a). Studies are especially concerned with analyzing the significance of (hetero-)sexuality in the construction of gender dichotomies and hierarchical gender relations in PE. They show that LGBTIQ students often face problems of being excluded, insulted, or attacked (e.g. Pérez-Samaniego et al., 2016; Müller and Böhlke, 2021) in PE, which is characterized by “narrow and defined norms of gender [and] sexuality and the body”^1^ (Piedra et al., 2016, p. 117). In this regard, LGBTIQ individuals or queer bodies in PE are often theorized as abject or oppressed (Pérez-Samaniego et al., 2016). Many queer educators and students still feel uncomfortable and limited in schools, despite progress in the acceptance of queerness in Western culture slowly making its way into schools (e.g., Ferfolja and Ullman, 2020). Particularly problematic practices in PE include dancing, changing rooms and restrooms as well as swimming (Landi, 2019a; Kosciw et al., 2020). Recent studies (Devís-Devís et al., 2018; Berg and Kokkonen, 2022) support the notion that traditional PE settings often support heteronormativity. Yet, they also show that PE can offer spaces to potentially challenge this and thereby provide positive experiences for queer individuals.

Beyond research on these aspects, there is a small number of studies that focuses on desire in PE. In her research on experiences of PE teachers, Sykes (2003) explores topics such as homoerotic desire between lesbian teachers and students. She describes the active suppression and concealment of desire by the individuals themselves, with the goal of fitting into the heteronormative system of PE. An example of this is pretending to be heterosexual, which is discussed by Landi (2019a,b) in relation to the central role of the body in physical education. Focusing on the experiences of queer male students, he emphasizes the active role of the field of PE in the production (not just suppression) of queer desire. PE materially produces and shapes such desires. Thus, there is a mutual influence between queer desire and practices within the structures of this field. This includes discussions about changing rooms as ambivalent homoerotic spaces that evoke both discomfort and erotic desire in queer students, as they involuntarily must deal with phenomena of physiological arousal such as erections (Landi, 2019a).

Building on existing research, critical pedagogical scholarship discusses PE as a learning space in which students of all sexualities can be sensitized and empowered in a unique way. Fitzpatrick and McGlashan (2016) criticize the dominance of a “straight pedagogy,” where heterosexuality is assumed, and they call for a radical rethinking of PE teacher education. Other scholars also propose pedagogical implications aimed at disrupting the field, with the overarching goal of challenging narrow and restrictive norms of gender, sexuality, and the body by conveying critical perspectives on health and physical activity (Larsson et al., 2014; Landi, 2018). Aspects such as the role of the teacher and the inclusion of or targeted focus on LGBTIQ students are discussed as particularly relevant in this context.

In sum, existing scholarship provides important empirical insights into the social construction of sexuality in PE while being mainly concerned with heteronormativity and LGBTIQ issues, yielding approaches for disrupting heteronormative structures within critical pedagogies. Studies about sexuality in general are lacking. In a previous study by two of the authors of this paper (Böhlke and Zander, 2022), emerging online spaces could be identified as arenas where current and former actors negotiate sexuality in PE more broadly, thus providing a promising research field to explore the phenomenon of sexuality in PE more generally. Focusing on students as central actors of PE and following a discourse theoretical perspective, our study is thus concerned with exploring the underlying patterns that structure the discursive construction of the phenomenon of students’ sexuality in online forums.

### 2.2 Theoretical background: sexuality as a broad field of research

Sexuality can be defined as a bio-psycho-social field of research (Dekker, 2013). There is a multitude of scientific perspectives (e.g., medical, biological, psychoanalytical, evolutionary psychological, social science) that draw on divers conceptual approaches and theoretical traditions to investigate sexuality not only as the reproduction of living beings but also with regard to aspects such as expressions of desire, relationship practices or forms of staging gendered bodies (Löw, 2008). In sometimes controversial debates, various explanatory approaches face each other, with attempts being made to find an approach that unites the disciplinary perspectives (Dannecker, 2017). In an interdisciplinary dialog, it is argued that physicality and sociality should no longer be analytically separated in terms of their influence on sexuality but should be more consistently related to each other (Dekker, 2013). A discourse-analytical approach can reveal which interpretations, perspectives and positions regarding the phenomenon of sexuality are taken up in what manner in a specific discourse field or discourse space.

#### 2.2.1 The discursive construction of sexuality

Rather than following essentialist notions, a discourse analytical perspective considers sexuality, sexual bodies, feelings, behaviors, and identities as being continuously constructed in socio-cultural practices and processes. In these socio-cultural practices and processes, discursively disseminated knowledge and offers of interpretation are drawn upon. Following Foucault (1983), sexuality can be understood as a phenomenon that emerges in discourses in a historically and socio-culturally specific form. This form makes it possible to “combine anatomical elements, biological functions, behaviors, sensations, and pleasures into an artificial unity and to allow this fictitious unity to function as a causal principle, as an omnipresent meaning and mystery to be decoded everywhere” (Foucault, 1983, pp. 148–149). Discourses thus produce ideas about multifaceted aspects such as anatomy, behavior, feelings or desires as something that belongs together. They relate these ideas to concepts of normality and morality (e.g., of certain sexual practices or orientations) as well as concepts of the functions of sexual desire (e.g., reproduction or relationship building). These ideas, concepts, and their relations are discursive knowledge constructions that function as generators and stabilizers of everyday actions and ways of interpreting oneself and others.

#### 2.2.2 Interpretative schemes

To investigate this relation between discourses and everyday ways of interpreting certain actions, phenomena, oneself, and others, our analysis focuses on the interpretative schemes that structure online communication on students’ sexuality in PE. Following the sociology of knowledge approach to discourse according to Keller (2013; Keller, 2018), the concept of interpretive schemes (in German: Deutungsmuster) refers to “social/collective meaning and action-organizing schemata, which are combined in and circulated through discourses” (Keller, 2018, p. 32). Interpretative schemes describe how people refer to discourses by interpreting specific everyday situations, actions, or phenomena in particular ways: “This concept has a particular importance for the relation between discourses and our everyday practices and self-understanding” (Keller, 2018, p. 33). Our research interest is to reconstruct which general patterns of interpretation – i.e., interpretative schemes – underly and structure online discussions about students’ sexuality in PE. Based on Keller (2013), our analysis was guided by the following sub-questions:

- *Fundamental idea of sexuality*: Which reference topics and fundamental ideas of interpreting students’ sexuality in PE are articulated in internet forums?
- *Manifestations of students’ sexuality in PE*: How are reference topics and fundamental ideas of interpreting students’ sexuality linked to (which?) everyday phenomena and problems in PE?
- *Dealing with students’ sexuality in PE*: Which ways, values, and norms of dealing with everyday phenomena and problems of students’ sexuality in PE are articulated?
- *Social differentiation of students*: Which social differentiations of students (e.g., in terms of gender or body) are made within articulations of ways, norms, and values of dealing with sexuality in PE?

## 3 Methods

### 3.1 Internet forums

Internet forums are online spaces for asynchronous (and mostly text-based) communication. Communication is initiated in a post about a certain topic and that post is then followed chronologically by responding posts, which often refer to and/or build on each other. This results in thematic sequences of communication, which are called threads. The users participating in this communication can choose their username and the personal information they want to convey, thus giving them the opportunity to also post anonymously. Because of that, internet forums can provide low-threshold spaces to connect with peers but also gain access to expert knowledge (Döring, 2013). Notably, expertise, just like other personal information and characteristics, is defined according to the information the users themselves share, which is rarely verified independently. Previous research has found that sexuality in PE is discussed openly and broadly in internet forums (Böhlke and Zander, 2022). From our discourse theoretical perspective, internet forums thus present a field of research that is suited for exploring the collectively shared schemes of interpretation that constitute the sexuality of students in PE as a discourse phenomenon. We consider internet forums to be a discourse space in which users through their posts engage in social processes of interpretative meaning-making that discursively construct students’ sexuality in PE according to such schemes of interpretation.

### 3.2 Data collection

Our study draws on a sample of 17 threads from the period 2015–2022 about students’ sexuality in PE from seven different German-speaking internet forums (Table 1). For generating this sample, we systematically considered relevant methodological literature (Holtz et al., 2012; Smedley and Coulson, 2021), carefully documenting and continuously reflecting our own strategies (e.g., with regard to relevant selection criteria of forums, threads and posts). Our data collection followed a phenomenon-oriented theoretical sampling strategy, which is appropriate for exploratory research aimed at developing a local theory about a specific phenomenon directly from the data. Theoretical sampling methods are originally situated in Grounded Theory Methodology (Glaser and Strauss, 1998, p. 53) but have since been adapted in other qualitative research approaches, including the sociology of knowledge approach to discourse (Keller, 2013) that our study draws on. Following this method, data collection was part of an ongoing iterative process in which the analysis of available data informed the collection of new data, the comparative analysis of which led to a richer and deeper understanding of the phenomenon. Specifically, our aim was to compile a sample whose threats and posts covered a maximum of different topics, aspects, and facets of the phenomenon of students’ sexuality in PE. Drawing on the existing research outlined above and particularly on a previous study on sexuality in internet forums (Böhlke and Zander, 2022), we searched various internet forums with search strings that combined PE-related keywords (e.g., physical education, sports, school, teacher, student) with keywords related to aspects of sexuality (e.g., touching, relationship, boobs, erection, aroused, horny). The keywords and search strings evolved during the iterative research process. We included all threats/posts that dealt with aspects related to students’ sexuality in PE and that met our ethical considerations (see below). All other threats/posts that did not meet these criteria were excluded. Our sampling process led us to identify six topics that most online discussions about students’ sexuality in PE revolved around (Table 1): desiring the PE teacher; erotic peer relationships; erotically connoted behavior of the teacher; nudity in changing room and showers; skimpy clothing; visible arousal. We looked to include threats in which these topics were discussed from different perspectives, that is, from the point of view of students, teachers, and parents (according to the users’ self-presentation), ceasing data collection when the variation of these perspectives and the criteria mentioned before was saturated. We pasted the threads into Word documents, saved screenshots to capture the visual elements of the websites, and wrote memos about our initial observations.

**Table 1:** Data corpus.

Topics	Thread	Contributions	Period (year)	Forum/Website
Desiring the PE teacher	In love with my (almost) former teacher	277	2022	Website A
	In love with hot PE teacher	10	2016–2020	Website B
	How to attract glances from my PE teacher?	12	2015–2016	Website B
Erotic peer relationships	Touch BFF in locker room ok?	31	2020–2022	Website C
	Getting boys hot in gym class?	6	2017	Website B
	Gay when I look at classmates’ underpants?	10	2017	Website B
Erotically connoted behavior of the teacher	Where is a PE teacher allowed to touch a female student?	14	2018	Website B
	Sexual harassment of teacher?	16	2017–2019	Website B
	My teacher is grabbing me!	12	2013–2016	Website B
Nudity in changing room and showers	In locker room pants down	44	2015	Website D
	Showering after PE and changing clothes	36	2010	Website E
	Swimming lessons and supervision in the locker room	58	2022	Website F
	Showering and changing with classmates	11	2018–2020	Website G
Skimpy clothing	Short tight sports pants too cheap?	13	2016–2022	Website B
	Tight leggings and a belly in gym class?	9	2017–2018	Website B
Visible arousal	Boner in PE, what to do?	8	2020	Website B
	How do you feel about seeing a boy in gym class with a stiffy?	23	2011–2019	Website B

All thread titles were translated from German by the authors and modified for the purpose of anonymization.

### 3.3 Data analysis

Our analysis was concerned with reconstructing the collectively shared interpretative schemes that underly the online communication about central topics of students’ sexuality in PE. Following the sociology of knowledge approach to discourse analysis according to Keller (2013, 2018), we analyzed the data material using two procedures. First, we conducted open coding of all data material. Second, we conducted sequential analyses of passages that we identified as particularly rich and relevant. Both procedures were guided by the sub-questions for reconstructing interpretative schemes that we presented in the theory section above.

### 3.4 Ethics statement

Based on recent discussions (Eysenbach and Till, 2001; Smithson, 2015; Schmidt-Lux and Wohlrab-Sahr, 2020) and in adherence to current guidelines (Franzke et al., 2020) on ethics in online research, our study followed a dynamic and situational approach to research ethics. In line with our sampling strategy presented above, this approach emphasizes an openly and reflexively designed research process in which ethical assessments and decisions must continuously take place as researchers gradually develop a deeper understanding of the characteristics of their online research field. Our study exclusively relies on internet forums that require no registration and are publicly accessible, justifying their consideration as “public behavior” (Holtz et al., 2012). This behavior happened without our interference as a form of ‘natural’ communication. Continuously deliberating this research field and the potential data material in our research team, we concluded that users in the online threads we included understand the public nature of their communication. They have moderate to low expectations of privacy, and they control which information to disclose, for example, through their chosen usernames or in their posts. Following the model for supporting ethical decision in (online) fieldwork by Heibges et al. (2019), we determined that, given these privacy expectations and characteristics of our research field, obtaining individual informed consent was not necessary. This decision is in line with recent discussions and guidelines on online research (e.g., Eysenbach and Till, 2001; Smithson, 2015; Franzke et al., 2020) as well as with existing studies (e.g., Lauritsalo et al., 2012; Sciberras and Tanner, 2023) that stress that informed consent can be waived if online communication is deemed public, the identity of the users can be protected and the potential for harm can be ruled out. To ensure the non-identifiability of individual users, safeguard personal rights, and prevent harm, we anonymized the data as required (e.g., removal of demographic or potentially identifying user data; omission of forum names in this publication) and maintained this anonymity throughout the research process. Moreover, we do not focus on individual persons and their personal experiences, as our discourse analytic research perspective is centered on collectively shared interpretative frameworks.

## 4 Results

Our analysis shows that students’ sexuality in PE is an ambiguous discourse phenomenon that is constructed in internet forums in a multifaceted way and leads to controversial discussions. However, we were able to reconstruct that this multifaceted online communication is grounded in and structured by two dominant interpretative schemes: The sexuality of students in PE is mostly interpreted as a phenomenon of biological development and as cultural phenomenon that is part of students’ identity. Notably, these two interpretative schemes offer different views on students’ sexuality in PE. Their common position is that both interpretative schemes recognize (from their respective point of view) the existence and the relevance of students’ sexuality in the PE classroom. Further, both view students’ sexuality as something that is subject to restrictive norms according to which it should be controlled or disciplined.

These results are now present in detail. We present them in an aggregated form that includes illustrative quotes from the data material. Our presentation starts out with a summary of the interpretative inventory of each scheme, which is then elaborated along our research sub-questions: What fundamental idea of sexuality is conveyed? How does the phenomenon of students’ sexuality manifest itself in PE classes? What are the ways, norms, and values of dealing with the everyday phenomena and problems of this manifestation? How are students socially differentiated in this context?

### 4.1 Students’ sexuality in PE as a phenomenon of biological development

In this discursive scheme of interpretation, sexuality is understood as a biologically determined developmental phenomenon. The posts that draw on this scheme tend to focus on bodily sensations and expressions of pleasure, desire, or arousal. They view these as biological facts, constructing students’ sexuality in terms of a human nature that must be understood and controlled.

#### 4.1.1 Basic idea of sexuality

Bodily sensations are the central reference theme of this interpretive scheme. They are interpreted as expressions of a sexuality that is located in the body. Viewed as a desire that manifests itself physically, these expressions are described in the posts as biologically regulated bodily processes. The body is thereby constructed as an anthropological fact imposed on all human beings, subject to age and gender. Underlying this notion is an assumption of a human being whose sexual acts serve a natural drive to procreate (“That man and woman are interested in each other was already the case with Adam and Eve!”). Further, this interpretive scheme contains the notion of puberty as a peak phase of sexual development in which the superiority of the body over the mind intensifies (“if something moves in the pants of boys in puberty, then it is simply natural”). Moreover, sexuality is not only interpreted as a phenomenon expressed through bodily sensations but also as exerting an influence on the body. This shows in posts that characterize puberty as a phase in which the development of sexuality leads students to examine their own bodies and the bodies of others anew. Additionally, posts that draw on this interpretive scheme refer to quasi-objective (technical) knowledge about human biology regarding the body and human sexual drive (“reason for spontaneous erections are hormonal changes”).

#### 4.1.2 Manifestations of students’ sexuality in PE

The specific actions and phenomena that are interpreted as manifestations of students’ sexuality in everyday PE classes include flirting, covert but also undisguised sexualized actions such as glances or touching as well as bodily signs of sexual arousal such as a “boner” becoming visible in shorts. In posts that draw on this interpretative scheme, these actions and phenomena are naturalized, e.g., by being declared to be expressions of a natural sexual drive that students are almost powerless against. PE is constructed as a field in which these expressions are provoked but also must be controlled.

Some posts, for example, describe that students at a particular age show a pronounced interest in the bodies of other students or teachers, or more specifically in their intimate body parts such as breasts, buttocks, or genitals. The posts describe that this becomes virulent or is even stimulated in characteristic situations of PE classes, such as situations where individuals present movements in front of others or in changing and showering situations. Some posts describe students engaging in body comparisons in these situations while others problematize the phenomenon of sexually motivated “gawking.” Most importantly, these actions and phenomena are naturalized and normalized as an age-specific search movement in the context of finding sexual identity or developing sexual interest: “Looking at other people’s butt is normal at your age - a lot of people do that to compare themselves with others,” “As a student, I could not take my eyes off the girls either!.” Underlying this interpretation is the idea that bodies react to other bodies quasi-automatically through some sort of stimulus–response chains. This idea delegates sexuality and the primary responsibility for it to the realm of biology. At the same time, posts call on individuals to deal with this according to the social conventions and rules that apply in PE classes. Thus, the central problem that is negotiated within this interpretative scheme is the necessity and, at the same time, the limited possibilities to control one’s bodily sexual impulses and expressions.

#### 4.1.3 Dealing with students’ sexuality in PE

Possibilities for dealing with this central problem are discussed controversially in the internet forums within a framework of partly contradictory values and norms. One dominant norm is the shifting of sexuality into the private sphere. Sexuality is declared to be an “intimate private matter” that is inappropriate in the public sphere of PE. This norm is expressed, for instance, in calls for refraining from sexualized acts (“In PE, it’s about doing sports and nothing else”) or for hiding signs of sexual arousal (“Just put on a tight pair of underpants under your boxer shorts, this will prevent your pants dancer from going upwards”). These calls for discretion transport the overarching goal of not appearing as a sexually active being to others. Failing to be discrete is depicted as a disruptive factor in the lessons and as an unacceptable act of (violent/powerful) transgression of the privacy of others. In accordance with this norm, persons that publicly exhibit sexuality in PE are called disgusting, perverted, or encroaching on others: “Disgusting, especially if he still thinks it’s cool to have a hard-on.”

This norm of privacy and discretion is, however, not absolute. Rather, the discursive inventory of this interpretive scheme contains different relativizations of this norm and even counter-norms. For example, responding to the question if it is appropriate to look at the naked upper body of others in the locker room, a user states: “Of course, looking at it intensively and deliberately would be more conspicuous, but if it’s inconspicuous, it’s okay.” In the public sphere of the locker room, looking – as a form of acting on one’s sexual impulses – is declared acceptable if it is done secretly enough. A different example are repeated calls for mildness and relaxation: “He cannot help it [erection during swimming lessons] and it’s already embarrassing for him. I would not make a drama out of it.” Drawing on the notion of expressions of sexuality (here: an erection during swimming lessons) being natural biological reactions, students are (partly) relieved of the responsibly to control them, and others are advised to tolerate or ignore them. Departing from this notion as well, few posts also encourage students to handle their sexually acting bodies self-confidently: “Wear it [the erection] with pride! The girls may giggle, but of course they like to see something like that.” Within the interpretative scheme, counter norms like this, which distance themselves from the norm of privacy and discretion, at the same time stabilize the dominant interpretation of sexual acts by students being considered failures of self-control and therefore embarrassing, cringeworthy, and out of place in PE classes.

#### 4.1.4 Social differentiations of students

The social differentiations that are produced discursively within this interpretive scheme construct sexuality, sex, and gender as an inseparable constellation. When addressing the phenomena, problems, and norms of students’ sexuality just described, many posts differentiate between students according to their sex/gender. Thereby, they (re-)produce specific gendered power relations. For example, this interpretative scheme contains the idea that pubescent boys are more strongly bound to sexual drives compared to girls. They are pictured as “testosterone-controlled” boys whose sexual receptivity poses a problem for PE classes. According to this interpretation, phenomena such as being aroused by others to the point where you cause distractions in the lesson are viewed as being a male problem: “Boys are not easy to handle in puberty in the presence of girls (...) especially in PE, when girls dress rather skimpily.” Girls, on the other hand, are predominantly positioned as causes of boys’ arousal and distraction, and they are at times addressed as being responsible for limiting these effects, e.g., through their clothing choices. Additionally, female students are positioned as victims vis-à-vis “libidinously” acting boys/men. A different example of social differentiations along the category gender, which are part of the inventory of this interpretative scheme, is that female students are attributed certain traits, such as being naturally oversensitive. They are assumed to prematurely interpret certain physical actions by classmates or teachers as sexual, particularly at a certain age: “Are you in middle school? That’s where female students see this kind of thing particularly often, sometimes unjustly.” As this quote illustrates, this can result in accusatory posts that downplay actions that were perceived as inappropriate. Other posts also open avenues for female students to deconstruct or invert these gendered power relations. They discuss possibilities for girls to use male sexual receptivity for their own purposes, for instance, by influencing a teacher’s grading through a revealing appearance: “What can I wear to get more attention from my PE teacher?” While opening different subject positions for female students, these posts still adhere to the interpretative scheme of viewing sexuality as a natural-biological phenomenon whose bodily impulses can be expressed and acted upon differently by males and females.

### 4.2 Sexuality as a cultural phenomenon that is part of students’ identity

The second interpretative scheme offers a fundamentally different discursive inventory. In this scheme, sexuality is understood as a cultural, socio-historically variable phenomenon that is part of student’s identity. As such, sexuality is not conceived as stable and singular but rather as a spectrum of sexualities that people choose from and actively shape.

#### 4.2.1 Basic idea of sexuality

Erotic preferences, sexual interests, and subjective forms of desire are the central reference themes within this interpretative scheme. They are discussed with regard to individual intentions, attitudes, and ideas that inform sexually motivated practices but also with regard to social conditions. Posts that draw on this scheme of interpretation associate sexuality with aspects of self-determination or freedom of choice within a given diversity of options, for instance regarding forms of desire, relationship constellations, and sexual practices: “I’m gay and I find my classmates’ upper bodies interesting, what difference does it make? 😊.” Additionally, such posts draw on publicly disseminated knowledge about (youth) trends, dress codes (“Belly-free is the trend today after all!),” body and gender politics as well as discourses about the current state of society. The central idea is that people, specifically students, actively (co-)construct, shape, and also control their sexuality as part of a self-determinant and self-responsible lifestyle in modern society.

#### 4.2.2 Manifestations of students’ sexuality in PE

Practices of bodily self-staging, desire in an overarching sense (e.g., sexually motivated behaviors in movement situations, glances at intimate body parts), and relationships in PE (e.g., student-teacher) are manifestations of students’ sexuality in PE that are discussed within this interpretative scheme. For instance, PE is associated with a particular type of clothing (lightweight, freedom of movement) that is discussed with regard to issues of bodily exposure. Several posts discuss the possibilities of students, especially female ones, deliberately staging themselves physically in front of others to be perceived as attractive or “sexy,” for instance by wearing hot pants or crop tops. The discussions revolve around the legitimacy of this self-staging. In some posts it is encouraged and viewed as a modern self-confident approach to one’s body and sexuality: “Do it! Quietly play with the charms and enjoy the looks,” “Come on, we live in the 20th century! It should be okay to wear belly-free.” In other posts, physically revealing self-portrayal is negotiated as an everyday beauty code with clothing being interpreted as a sign of one’s own youth cultural positioning: “Stay cool and wear what you like, as long as the teachers do not say anything.” Again, other posts feature derogating comments about females engaging in this self-staging, especially if they explicitly express the intention of attracting others’ attention: “Do you want to look like a bitch?.” However, such defamation is also called out as “slut shaming.”

The central problem that is discussed is how to deal with the diversity of sexuality-related options that students face in different situations. Some posts cover fundamental questions, e.g., about sexual orientation, while others touch on topics such as belonging to a certain social group, for instance the “mature” that engage in sexual relationships vs. the “young ones” that are not interested in sexuality. These phenomena and issues are not discussed from an “anything goes” perspective but rather against the backdrop of specific notions of legitimacy and particular moral concepts that have a strong normative impact.

#### 4.2.3 Dealing with students’ sexuality in PE

In this interpretative scheme, the dominant norm for dealing with students’ sexuality in PE refers to a notion of school as an asexual place. Actors in school are reduced to their roles as teachers and students, with the expectation that they should subordinate their personal preferences and qualities to these roles. This specifically includes the expectation of self-regulating one’s sexuality, as any kind of sexuality – except for sex education work – is seen as not belonging in school. This regulative norm is articulated, for instance, through posts that interpret sexually motivated acts in PE as inappropriate: “He [student who lets his pants down in PE class] has to behave appropriately in public and refrain from doing that.” Other posts assert normative truths about sexual relationships, citing the institutional roles of the individuals involved. As a result, the possibility of discussing such matters in online forums is consistently dismissed in such posts: “Teacher with student does not work. End.” According to this norm, teachers’ actions toward students that are perceived as sexual, such as physical touching of intimate body parts during assistance, are unacceptable: “Report immediately! Pedos do not belong in school!” In other posts, this norm of self-regulation is expressed with regard to students’ clothing choices: “If I were you, I’d rather wear something discreet in PE!” The morality and character of individuals who do not adhere to the notion of school and PE as sexuality-free spaces are judged accordingly: “I think girls who come to PE with a deep neckline are just bxxx.” This norm may seem similar to the calls for decency and self-control mentioned in the context of the first interpretative scheme above. However, the posts mentioned above draw on the notion of sexuality as natural and biological impulses that should be controlled. In contrast, when analyzed in their discursive context, the posts mentioned here draw on a notion of sexuality as the free choice and self-determined expression of sexual identity, which should not be practiced in school or during PE.

#### 4.2.4 Social differentiations of students

The manifestations and norms of students’ sexuality in PE that shape the discursive inventory of this interpretative scheme lead to different social differentiations. One is the difference between hetero- and homosexuality. Several posts specifically deal with issues of homosexuality in PE, for instance an interest in the bodies of same-sex classmates. These issues are often normalized or even prioritized with reference to developments toward openness and sexual diversity in society: “Being gay is completely normal today.” Secondly, as already mentioned above, actors are differentiated according to their institutional roles in PE. Choosing, exploring, and expressing one’s sexual identity is discussed differently for students and teachers. For teachers, the norm of keeping sexuality out of PE is made uncompromisingly binding, with posts referring to role-specific requirements such as the duty of care and ethical considerations, for instance, regarding age differences. Thirdly, gender related differentiations come into play again. These touch on issues also mentioned in the presentation of the first interpretative scheme above, such as a presumed heightened sensitivity of female students. Here, this sensitivity is interpreted to be a social rather than a natural-developmental phenomenon, for instance by being attributed to social movements in current society: “One wrong saying and you are already a pervert as a man today. Just because this stupid me-too movement is trendy these days.” In these discussions, supposedly over-sensitive female students are, on the one hand, called upon to question their assessments or to refrain from rash actions such as publicly accusing a teacher. On the other hand, the discussions, among others, also feature constant appeals to girls to defend themselves, communicate their discomfort to others, and empower each other in the sense of female self-emancipation (“Defend yourself! Your teacher has no right to grab you at any time”).

These examples show how controversial discussions and very different positions in internet forums about sexual orientations, roles, and gender relations in PE draw on a common, underlying notion of sexuality as an elementary and omnipresent feature of adolescent life. Within this interpretative scheme, skillful handling of this feature ensures social acceptance among peers by creating group affiliations and showing boundaries, e.g., by illustrating a modern way of thinking.

## 5 Discussion

From a discourse analytical perspective, sexuality is a multifaceted phenomenon that is shaped, de- and reconstructed in various discourses of modern societies. In this paper, we employed this perspective to conceive of PE as a field in which such discourses intersect to help inform the practices, experiences, and realities of this field. We identified internet forums as spaces in which active negotiation and (re-)production of discourses on sexuality in PE happen intensely and broadly. Focusing on the sexuality of students in PE, our study was interested in exploring which dominant schemes of interpretation underly and structure the negotiations of this phenomenon in German-speaking internet forums.

Our study reconstructed two dominant schemes of interpreting students’ sexuality in PE, both of which are differentiated in complex ways. Our main finding is that discursive constructions of students’ sexuality in PE predominantly draw on – and by that reproduce – a ‘nature’ and a ‘culture’ perspective on constellations of body, sex, gender, and sexuality. That is, the interpretative schemes we have identified reflect the central perspectives of biological-natural scientific as well as cultural and social scientific approaches (e.g., Wrede, 2000; Dannecker, 2017) that traditionally structure the sexual science discourse. While these perspectives typically exist independently within different disciplinary communities and are often placed in opposition to one another (e.g., Benkel and Lewandowski, 2021), our study demonstrates that in everyday online discourse, individual interpretations and arguments from each perspective are taken up and related to each other in complex ways. Within the online threats, each perspective features specific knowledge about human nature, the body, adolescence, or PE under current social conditions. From each perspective, different everyday phenomena and problems in PE and different norms for dealing with these phenomena and problems become important. Similar phenomena, such as the presumed heightened sensitivity of female students, and norms, such as a call for self-regulation, are interpreted and explained differently. Many posts draw on a ‘nature’ or ‘culture’ perspective with absolute claims to truth, devaluing or delegitimizing post and arguments from the other perspective. At the same time, other posts do not adhere to this binary logic. Given the multifaceted and complex nature of the online discussions about students’ sexuality in PE, our findings thus provide a better understanding of the overall discursive context and the basic interpretative inventory in which individual posts or articulations in internet forums are situated, and they reveal these online discussions as a site of cultural wrestles over the dominance of certain forms of knowledge and subjectivity between ‘nature” and “culture” two schemes of interpretating sexuality.

Previous research shows that social constructions of sexuality in sport continue to reinforce binary and heteronormative discourses (Sykes, 2003; Fitzpatrick and McGlashan, 2016; Landi, 2019a,b), with heteronormative ideas being defined in particular by the naturalization of heterosexuality and dichotomous gender. Our study confirms this to some degree regarding the social constructions of students’ sexuality in PE in internet forums. Both reconstructed interpretative schemes provide knowledge about manifestations of “male” and “female” sexuality of students in PE that supports heteronormative views. The “nature” scheme of interpretation, for instance, draws on a quasi-objective knowledge about the human body and its biology, e.g., regarding male students’ sexual drive. The “culture” scheme of interpretation draws on knowledge, e.g., about the social production of female heightened sensitivity as a current social phenomenon. However, especially the ‘culture’ scheme of interpretation features knowledge and “truths”, e.g., about choosing and expressing sexual preferences autonomously, that also offer non-binary and non-heteronormative perspectives.

Another aspect discussed in existing research is the elimination of sexuality from PE (Sykes, 2003; Landi, 2019a). Sykes describes sexual desire between students and teachers as a “trope of silence” (Sykes, 2001, p. 14) and states that, in the heteronormative context of PE, teachers can only succeed if they adhere to the normative expectation of suppressing or concealing desire. Our study confirms these results regarding online communication about students’ sexuality in PE, and it expands on them by revealing their constitutive discursive context und underlying interpretative schemes. From their respective perspectives, both interpretative schemes work on eliminating sexuality from PE. While they explain and locate sexuality differently (nature vs. culture, bodily impulses vs. personal choice), they both take the individuals to be responsible for keeping sexuality out of PE. For this, sexuality is perceived as a private matter in the naturalistic perspective, while in a sociocultural perspective, it is tabooed within the context of institutional roles and relationships. Additionally, we were able to surface that these discursive schemes also offer possibilities to counter or relativize this norm of silence. For example, in the “nature” scheme, it is assumed that students have limited ability to control their pubescent bodies, while in the “culture” scheme, sexualized self-staging through wearing revealing clothes is legitimated with common clothing styles in (western) modern societies.

The limitations of our study pertain to its focus on the German-speaking context, on students’ sexuality, on internet forums, and on current PE-related communication. Since the social construction of sexuality is always situated in a specific cultural and societal context, in which it is specifically positioned (Fitzpatrick and McGlashan, 2016), future research should include online forums from other national school systems and cultural contexts. It should take the phenomenon of teachers’ sexuality more prominently into account. It should investigate other discourse spaces, both online and offline, with the aim of mapping what is and can be said about sexuality in PE by whom and in which spaces. It should trace in more detail which larger discourses are referenced or cited. One possibility would be following up with a survey or face to face interviews. Additionally, further research should also take a historical perspective to reconstruct sexuality in PE as a socio-historical phenomenon. Our discourse analytical focus on interpretative schemes and the two interpretative schemes that we were able to reconstruct in this study may serve as important reference points for such future research. Focusing on specific phenomena of sexuality in PE, such as homoerotic desire, being in love with the teacher, or clothing styles in PE, future research could investigate how “nature” and “culture” interpretations are actually invoked and (inter-)related in discourses on these phenomena.

For pedagogical practice, our study suggests that it is important to address sexuality in a subject-specific approach and take the discursive knowledge and fundamental patterns of interpretation into account that shape the (im-)possibilities of addressing sexuality in PE. As a school subject, PE features several characteristics that distinguish it from other subjects, chief among them its pronounced focus on the body (Landi, 2019a; Berg and Kokkonen, 2022). This makes PE a very specific field for students and teachers to make sense of various facets of sexuality. Our study highlights that this sensemaking includes navigating different and partly conflicting knowledge, interpretations, and norms that converge in two basic interpretative schemes. Following Van der Steeg et al. (2021), we find it important to create ethical concepts of positive sexual integrity that not only protects students from harm, but also proactively contributes to healthy sexual development. We support existing sport pedagogical approaches that focus on questioning binary and limiting norms regarding gender, sexuality, and body to promote sexuality/gender/body-related diversity (e.g., Larsson et al., 2014), for example in the context of transformative pedagogies of physical education (Fitzpatrick and Enright, 2016).

Particularly, our study supports approaches that try to make PE a diversity-sensitive environment (Ruin and Stibbe, 2023) in which non-binary, non-heteronormative, inclusive interpretations of sexuality can be explored (e.g., Landi, 2018). This includes providing immediate support services for students and teachers who are vulnerable to (allegations of) sexual harassment, abuse, or exclusion, such as anonymous complaint channels or information and counseling services. In addition, long-term prevention measures are necessary, such as mandated workshops by external and explicitly trained sexual educators that could be incorporated into the curriculum or no-go and best-practice examples that are integrated into teacher training to enhance the awareness of current and future physical educators (e.g., Böhlke et al., 2022). Yet, pedagogical approaches should also go beyond a focus on negative phenomena such as harassment or exclusion and conceptualize PE as a space particularly suited for discussing topics such as desire, physicality, closeness, or intimacy and questions related to clothing, relationship building, or group belonging with students. This should also include reflecting on the discursive “truths” and patterns of interpretation that are reproduced or challenged in such discussions. After all, talking about sexuality is a powerful practice that shapes realities of PE.

## Data availability statement

The raw data supporting the conclusions of this article will be made available by the authors, without undue reservation.

## Author contributions

NB: Conceptualization, Data curation, Formal analysis, Funding acquisition, Investigation, Methodology, Project administration, Resources, Software, Supervision, Validation, Visualization, Writing – original draft, Writing – review & editing. BZ: Conceptualization, Data curation, Formal analysis, Funding acquisition, Investigation, Methodology, Project administration, Resources, Software, Supervision, Validation, Visualization, Writing – original draft, Writing – review & editing. DR: Conceptualization, Data curation, Formal analysis, Funding acquisition, Investigation, Methodology, Project administration, Resources, Software, Supervision, Validation, Visualization, Writing – original draft, Writing – review & editing.

## Footnotes

^1^All translations from German or other languages by the authors.
